# Evaluating the impacts of land use/land cover changes across topography against land surface temperature in Cameron Highlands

**DOI:** 10.1371/journal.pone.0252111

**Published:** 2021-05-21

**Authors:** Darren How Jin Aik, Mohd Hasmadi Ismail, Farrah Melissa Muharam, Mohamad Azani Alias

**Affiliations:** 1 Department of Forestry Science & Biodiversity, Faculty of Forestry and Environment, Universiti Putra Malaysia, Serdang, Selangor, Malaysia; 2 Geospatial Information Science Research Centre, Faculty of Engineering, Universiti Putra Malaysia, Serdang, Selangor, Malaysia; 3 Department of Agriculture Technology, Faculty of Agriculture, Universiti Putra Malaysia, Serdang, Selangor, Malaysia; Northeastern University (Shenyang China), CHINA

## Abstract

The Cameron Highlands has experienced multiple land encroachment activities and repeated deforestation, leading to extensive land-use and land-cover change (LULCC) during the past six decades. This study aims to determine the LULCC against topography in Cameron Highlands between 2009 and 2019 by using geospatial techniques to analyze Landsat 7 (ETM+) and 8 (OLI/TIRS), ASTER GDEM and MODIS imaging sensors. The results showed a decline of 35.98 km^2^ in primary forests over ten years across the Cameron Highlands, while agricultural lands and urban areas flourished by a rise of 51.61 km^2^ and 11.00 km^2^ respectively. It can be noted that the elevation most affected is between 1000 and 1500 m, across all classes. Further results showed the expansion of both agriculture and urban development onto slopes above 35°, leading to an instability of soil structure. In a comparison of the base years of 2009 with 2019, mean LST results have shown temperatures rising by 7.5°C, while an average between 3 and 4°C across the region is recorded. The results obtained provide new information for government bodies and land planners to coordinate their actions without further jeopardizing the environment of the Cameron Highlands.

## Introduction

The examination of land-use and land-cover change (LULCC) is a study of environmental change that is closely related to socioeconomic development. This change is mainly caused by the desire, rather than the need, to further expand land use, whether it be to satisfy agricultural or urban growth [1, 2]. Often, the expansion of land is improperly monitored with little or no consultation prior to the starts of projects. Thus, the use of various satellite datasets through remote sensing can provide a baseline requirement for sustainable planning and the management of natural resources [3–5].

In mountainous regions, any change in land use in the upper elevations will severely impact the overall climate of the region; This is because mountainous regions have a climate system that differs from the lower elevations; they are known to be highly susceptible to this change. This includes the study area of the Cameron Highlands [4, 6]. This region is regarded as an ideal area to study the effects on ecosystems because of LULCC. Due to their topography, mountainous regions are able to store and filter various energies, in addition to providing homes for a high diversity of organisms [1, 7]. According to Wang et al. [1], due to their “unique geology, geomorphology, climate, hydrology and other environmental characteristics, mountains gave birth to various ecosystems and provide diverse ecosystem services to local and lowland populations”. Although mountainous regions can provide resources to the local population, the expansion of industrialization and urbanization has pushed once lush forested areas to the brink of destruction [8, 9].

The relationship between LULCC and human growth for development is a complicated issue in Cameron Highlands, in addition to other mountainous regions in Malaysia, primarily due to the suitability of the climate for agricultural needs and to enhance local economic growth [4, 5, 10, 11]. The Cameron Highlands has a temperate climate with an average annual temperature of 23°C, thus prompting economic development in the 1970’s [12]. The area’s low and stable temperatures throughout the year, paired with a surrounding enclave of primary forests, meant that the area is ideal for the expansion of both tourism and agriculture [6, 13, 14]. The population growth has led to a rapid change in land-use and land cover (LULC) as a result of urbanization and industrialization. Although the changes in LULC do not directly imply a degradation of the land, under certain circumstances, improper handling of land use leads to a “landslide effect” that results in new problems. In the Himalayas, inappropriate deforestation and land use practices have led to accelerated erosion, which then further contributed to devastating floods in the lower plains [4]. Such disasters are common in Cameron Highlands [15]. For the past 30 years or longer, erosion, landslides, water table exposure, soil instability, and various other land failures have occurred as a result of improper land use and examination prior to a development [16].

Several studies have been conducted on LULCC in high elevation regions, with an emphasis on slopes [8, 17–19], mountains [19, 20], coastal regions [21–23], and elevated terrains of numerous landscape patterns [24, 25]. Landscape changes undoubtedly brings both positive and negative changes to an area [21]. In the Ganjingzi District, Yang J et al. [26] simulated a land use change in rural-urban areas. Using a four-part Markov Chain sub-area composite model, he found that the overall landscape conversion occurred in predominantly agricultural farmlands and garden lands, converted to construction land for urban expansion between 2000 and 2015. It can be noted that the use of this model can provide valuable management in land use planning. Wang et al. [8] studied land use change in the Tibetan plateau during the economic boom of 1990–2000. The results of his landscape metrics showed cropland and built-up land increasing severely, the magnitude of this change is driven by economic development. A land use assessment according to elevation and slope gradient was performed in the highlands of Ethiopia [18]. This study derived the use of Landsat imagery from 1986 to 2017 in the agricultural region of Amhara State. The study notes that the majority of land cover change is contributed not only by farmland expansion but also through illegal deforestation for logging exports. Furthermore, Chen et al. [19] used spectral indices to map landscape types of the mountainous region of Shizhu county. Similarly in a mountain range, the Fanjingshan Nature Reserve in China as an example, Tsai et al. [20] used a number of satellite sensors including Landsat, Quickbird and GeoEye to monitor land use changes in the national park. Their research showed the use of multi-temporal satellite imagery across multiple sensors, combined with techniques of seasonal image mosaics, digitized terrain models, and in-situ data to be of utmost importance in improving mapping accuracy. An assessment between the relationship of LST and LULCC was performed in Dongting Lake, China [27]. Tan et al. [27] found the external activities such as human induced activities, airflow, precipitation, and landscape pattern affecting the overall LST value. Rodrigues-Galiano & Chica-Olmo [22] analyzed a coastal area of Mediterranean Spain incorporating digital terrain models, land surface temperature and Landsat imagery over several seasons. Gaveau et al. [28] assessed deforestation in Borneo over four decades using Landsat, ALOS Palsar, and MODIS imagery. This research found native rainforests subjected to continuous logging, slash-and-burn practices, and conversion of land primary forest cover to industrial plantations. The studies above provided knowledgeable literature that is adaptive and facilitates the research background needed in the progression of methods. However, this study will be the first on the Cameron Highlands, focusing on the topographical classification (slope and elevation) of land use classes. This area has a landmass that is densely populated in the mid-elevation regions (montane forest oak of 1200 m), in which both agricultural and urban activities occur. Land cover changes in these areas for non-forested areas have increased by 161 km^2^ over the past 30 years, whereas other sources have reported an increase of 84 km^2^ during the same 30-year period [6, 29, 30]. Hence, this research is the first for the study area to use geospatial techniques to detect the LULC changes in Cameron Highlands in relation to topography from 2009 to 2019.

In this paper, we discuss on the following objectives: (i) Assessing land use and land cover change across elevation, (ii) classifying slope degrees according to their associated land use to assess the changes in land cover, (iii) analyzing LST patterns against topography (LST per elevation / LST per slope), (iv) identifying the driving forces of LULC in Cameron Highlands, and (v) consider methods of mitigating the driving forces to achieve sustainable development goals.

## Materials and methods

### Study area

**Fig 1:**
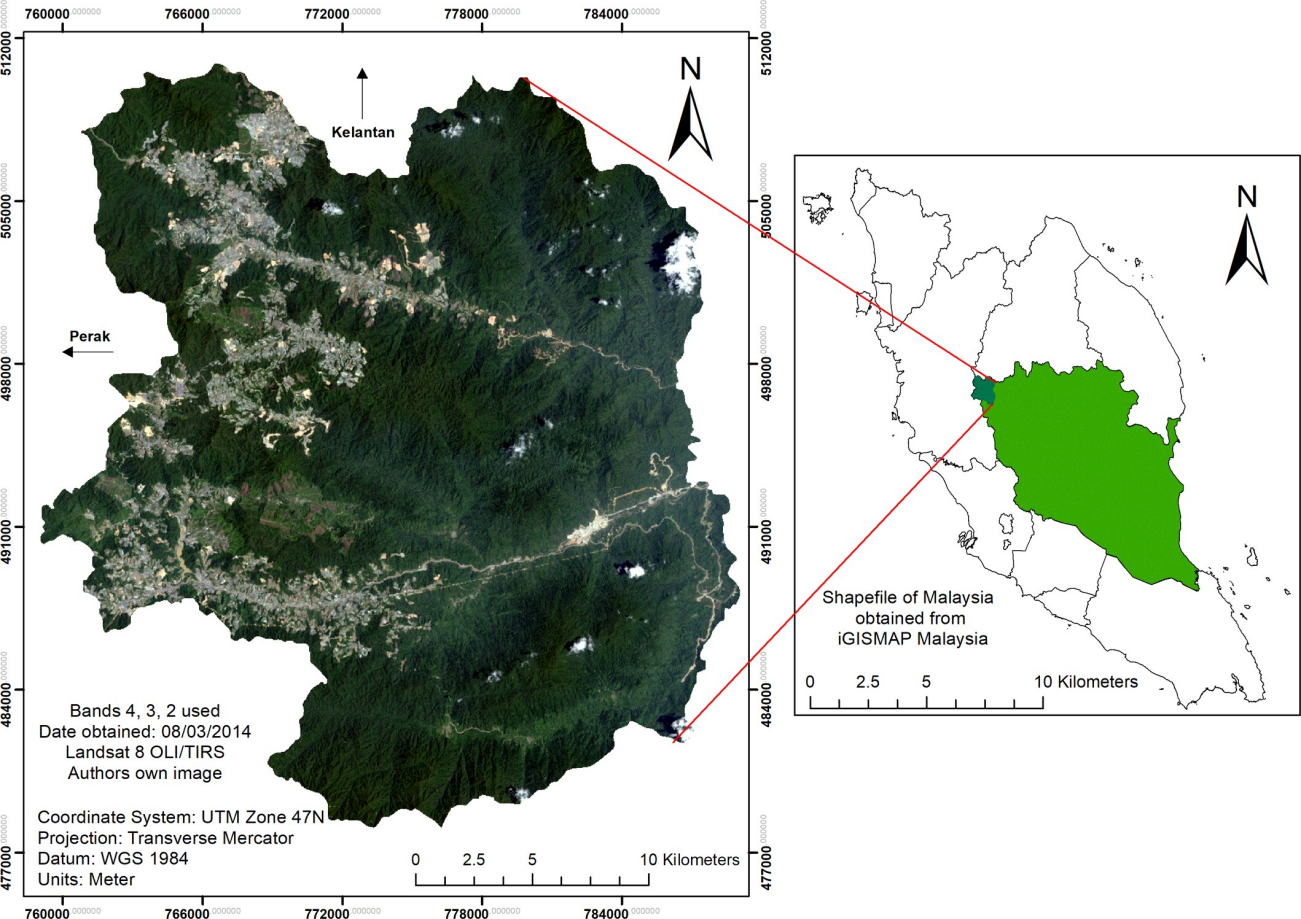
Study site of the Cameron Highlands District in the state of Pahang, Malaysia. (Data source: Landsat-8 OLI image courtesy of the U.S. Geological Survey).

This study was conducted in Cameron Highlands district, located in the western part of Pahang state, and defined as a highland area at 4° 35’ 55.40” N latitude and 101° 29’ 07.05” E longitude (Fig 1). Topographically, the study area contains roughly 50% mountainous areas, 30% undulating areas, 15% valleys, and 5% plains. The Cameron Highlands sits at an elevation between 300 and 2060 m above sea level, and its total area is approximately 69,699 km^2^. The soil structure classification of the area consists of sandy clay loam, limestone, and slate in the upper hill dipterocarp region and below (< 1200 m), as well as granite for the montane forest oak region and above (> 1200 m) [9, 31, 32].

### Background

Recently, the Cameron Highlands has been a popular research topic due to its numerous landslide accidents and sinkholes [29, 31, 33, 34]. The annual average rainfall in the area is 2850 mm. The combination of heavy precipitation and ongoing development of urban areas has led to the instability of the soil in the area. Moreover, the uprooting of forests and deep roots has also hastened the weakening of the structural integrity of the mountain. A guideline for land development according to slope degree was introduced by the authorities, it outlines the specific slope bracket in which certain types of land uses and construction are able to take place there [25]. However, contractors often defy the relevant regulations of that guideline and continue with their construction plans [25].

A preliminary study analyzing the connection between LULC and land surface temperature (LST) for the study area was extensively discussed by How Jin Aik et al. [35] using multi-temporal satellite images of Landsat 7 and 8 to provide a better understanding of the LULCC in the highland regions. The results illustrated an increment of total urban area by 1.7% in 10 years, while farmland and agriculture rose by 7.71% significantly. These changes to the land cover of Cameron Highlands had led to a decrease in primary forest cover by 8.88%. It was also recorded that temperature had risen between 2–3°C, with an extreme of 7°C on average during the period of the study. A similar approach was adopted for the current study to determine changes to the landscape and suitability of a specific land cover or land use-elevation and slope classification for future activities for the period of 2009 to 2019. An assessment of land surface temperature (LST) against topography of each land use class was also conducted for the same period. This research has identified the drivers of deforestation and established some recommendations for effective land conservation and management, as well as facilitate remote sensing technologies to provide a better understanding of the causes and consequences of land use change. The results of this paper can be used as a guideline for future studies to assess the relationship between LULC and LST in sensitive mountainous regions. Additionally, it could serve as a study on climate change as a result of deforestation projects.

### Data collection

Landsat satellite images from 2009, 2014, and 2019 (Enhanced Thematic Mapper+ ((ETM+) and Operational Land Imager/Thermal Infrared Sensor (OLI/TIRS)) were down-loaded from the United States Geological Survey Earth Explorer website (USGS) and used for this study. The Universal Transverse Mercator (UTM) projection of 47N and the World Geodetic Systems (WGS)–1984 datum was applied to the images. Landsat 7 was used for 2009, and Landsat 8 was used for 2014 and 2019. Images were processed into a level-one terrain-corrected (L1T) product. The Advanced Spaceborne Thermal Emission and Reflection Radiometer Global Digital Elevation Model (ASTER GDEM) was downloaded from NASA’s Earth Data website. The dates chosen for this study are in the first quarter of each year, this was to coincide with the ground-based data we had collected from a field visit at the end of March 2019 (Table 1).

**Table 1:** Description of satellite sensors and acquisition date.

Sensor	Date	Path/Row	Purpose
Landsat 7 (ETM+)	03 April 2009	127/57	Land Use vs Topography, Land Surface Temperature vs Topography
Landsat 8 (OLI/TIRS)	08 March 2014	127/57	Land Use vs Topography, Land Surface Temperature vs Topography
	22 March 2019	127/57	Land Use vs Topography, Land Surface Temperature Topography
ASTER GDEM V2	2011	ASTGTM2_N04E101	Digital Elevation Model derivation and Land Use vs Topography classification

### Image pre-processing

**Fig 2:**
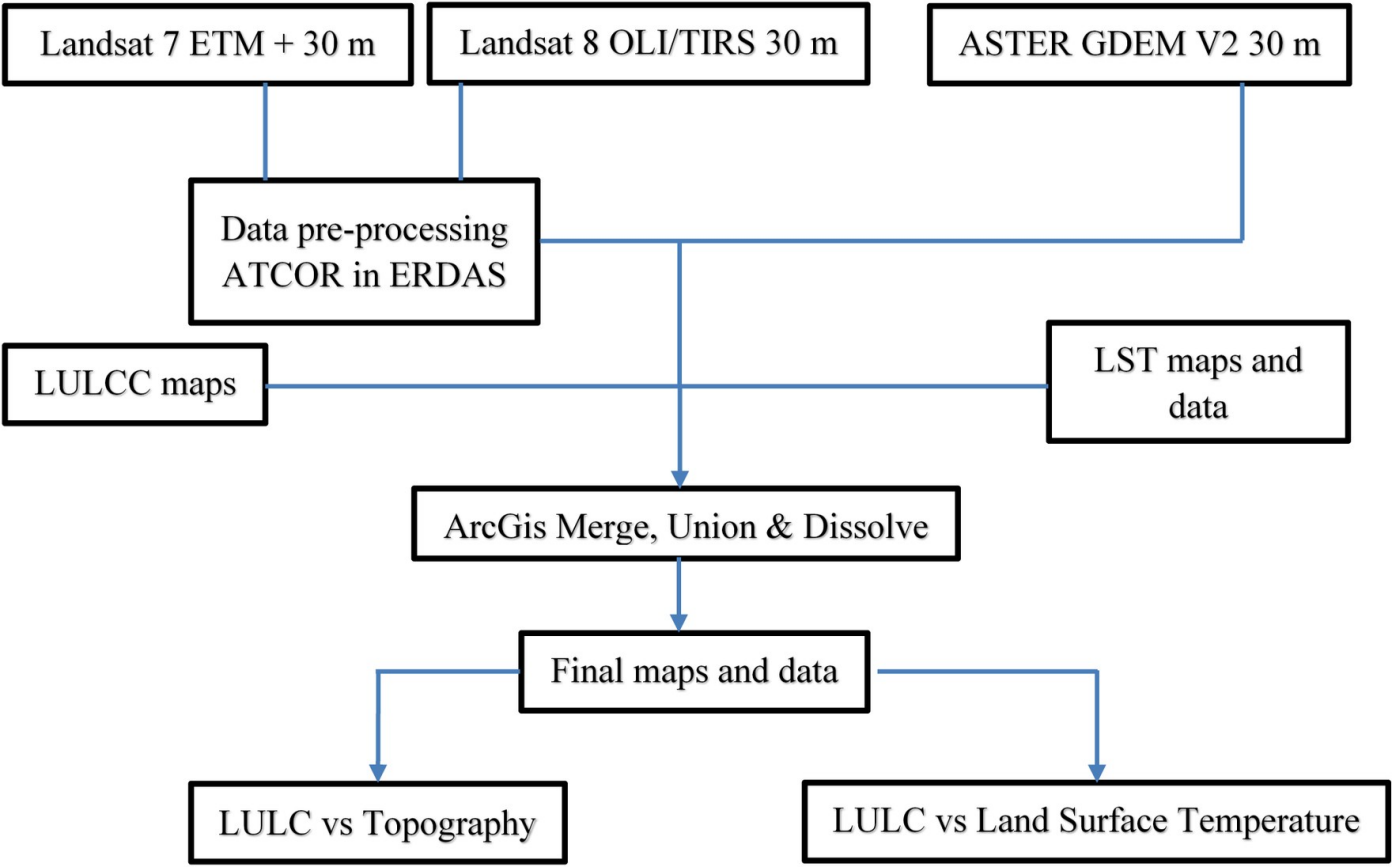
Flowchart outline of the study methodology.

Satellite images of the study years were imported into ArcMap 10.5 for mosaicking and data tiling. They were further imported into the ERDAS ER Mapper 2015 for additional image correction and cloud masking applications. In the ATCOR module, MODTRAN code was applied to the satellite images; this program proved useful in removing haze from the images. An outline of this process is shown in Fig 2.

### Land cover change across topography

#### LULCC and elevation

Because LULC is further classified according to elevation classes, the mountain range was divided into various subsections. First, the Global Digital Elevation Model was pro-processed and clipped to the study area. Then, the resulting image was reclassified according to the elevation, whereby 4 classes with an interval of 500 meters were obtained. The elevation interval was based on Meybeck et al. [36] typology grouping, where the mean medium of relief surface roughness, type of forests, and the grouping of urban areas are taken into consideration. The land cover classification image was then layered over the reclassified elevation map to be reprocessed. Thus, the newly classified image was then reclassified once more, resulting in a map of land cover against elevation groups. These elevation groups are listed as 1, 2, 3, 4, and 5 (< 500 m, 500–1000 m, 1000–1500 m, 1500–2000, and > 2000 m respectively). Using this final map, we were able to obtain land cover area values of the elevation groups. These processes were also repeated for the other years.

Finally, for the ground-truth images, a point-based accuracy assessment was con-ducted. On-site GPS points were obtained and applied on the 2019 imagery only, while Google Earth acted as an additional verification source for the other years. A confusion matrix was used in ArcMap to create 500 random sampling points, where 100 points were allocated to each land cover class. ArcMap was then used for the final stages of map making and labelling.

#### LULCC and slope

An additional step for assessing LULCC in the mountainous region used a slope degree map. The resulting image from the GDEM merger was used to produce a slope map originally derived by the unit degrees. Further, these degree values were reclassified to intervals of 10°. Similar to the methods conducted in the LULCC and elevation section, land cover classes intersected these degree lines were obtained. Finally, the land cover map was overlaid onto the slope map, and specific land cover types and their associated slope degree values with the resulting area values were obtained. The changes in slopes and elevation classes according to the LULC were tested using the PROC GLIM-MIX in SAS Ver. 9.3 (SAS Institute) at a significance level P < 0.05 as a split plot randomized complete block design (RCBD). The main plot was the land use classes, while the sub plot was either slope or elevation classes, while years served as the blocks.

### LST derivation

For the derivation of LST, we had used Landsat 7 and 8 TIRS bands with a thermal spatial resolution of 60 m and 100 m respectively. Landsat 7 was then resampled to the same spatial resolution of band 10 and 11 of Landsat 8. For MODIS, the spatial resolution is 1 km for bands 31 and 32; which was then reclassified to the same resolution as both Landsat 7 and 8 sensors– 100 m. As many dates throughout the months of March and April were shrouded in clouds, we had to go for the image with the least cloud coverage, then subsequently conducted a cloud removal technique in the ATCOR module of ERDAS Imagine 2015. Due to that, one image per month could only be obtained for all years of the duration of the study. The Landsat 8 satellite passed overhead our study area at approximately 10 am, consistently for all years. The temporal resolution for both Landsat 7 and 8 satellites is 16 days, while MODIS is 2 days. A known issue for Landsat 7 imagery was the SLC-off error, that removed important areas from the imagery, then, ‘gap filled’ using a gap filled data file that came within the Landsat imagery database.

The data used for our study are within the first quarter of each year (January to April). As the study area was known to contain plenty of cloud cover, due to it being a mountainous region that sits along the Titiwangsa Mountain range, it experiences harsh weather during the monsoon season. For this matter, MODIS was introduced to complement Landsat data as a means of validation to accurately estimate LST values. The MODIS data chosen was ensured to be a day or two before/after the images of Landsat. All data from the satellite sensors generated a maximum, minimum, average, and standard deviation values. These values were extracted from histogram tab in the data when viewed in ArcMap, for the purpose of LST analysis and map making accordingly. The LST derivation was conducted on the spatial scale of the entire study area– 669.69 km^2^, with and without land cover changes. Due to the varying LST values, the decision to combine Landsat and MODIS data was undertaken to obtain an average between the two sensors. Further explanations regarding the decisions above are covered in the preliminary study [35].

For this study, Landsat 7 ETM+ dual thermal bands were used; specifically, the low gain band 6 (6L) and high gain band 6 (6H). Band 6L shows areas where the surface brightness is high (non-vegetated areas), whereas band 6H highlights areas where surface brightness is low (vegetated areas) [27, 37]. In a study where the investigated area sat in a high vegetation area, Nguemhe Fils et al. [38] used only band 6H because the surface brightness was lower. However, in the current study, we used both bands because the study area comprised both vegetative and non-vegetative details. To obtain an average, both values of 6H and 6L were added and then divided by ‘2’. Various studies, such as [37, 39–41], previously used this method.

To calculate the LST of Landsat 8, we chose the Split-Window Algorithm (SWA), in which both bands 10 and 11 were applied; this method is recommended over other methods, despite the known stray light issue [42–48]. For the calculation of LST from the satellite data, the satellite images used were subjected to a series of processes. Additionally, MODIS TERRA, as a validation comparison, was also used within this study. The method of deriving LST is explained in detail by How Jin Aik et al. [35].

## Results

### LULCC accuracy

To calculate the accuracy of the classification and post classifications, a confusion matrix was performed using a point-based validation system. For the year 2019, ground truthing points collected on site by a handheld Garmin GPS is used for the accuracy assessment. However, as there are no user obtained points or historical classification maps for years 2009 and 2014, Google Earth acted as the validator. The results are shown in Tables 2–4. The overall accuracy was 94.60%, 90.94%, and 92.78% for years 2019, 2014 and 2009 respectively.

**Table 2:** Confusion matrix of 2019 land cover classification.

LULCC 2019	Primary	Agricultural	Urban	Cleared	Water	Total	User (%)
Primary Forests	97	4	2	0	0	103	94.17
Agricultural Lands	2	89	5	2	0	98	90.82
Urban Areas	1	7	92	3	0	103	89.32
Cleared Lands	0	0	1	95	0	96	98.96
Water bodies	0	0	0	0	100	100	100.00
Total	100	100	100	100	100	500	94.65
Producer’s Accuracy (%)	97.00	89.00	92.00	95.00	100.00	94.60	

**Table 3:** Confusion matrix of 2014 land cover classification.

LULCC 2014	Primary	Agricultural	Urban	Cleared	Water	Total	User (%)
Primary Forests	96	3	2	0	0	101	95.05
Agricultural Lands	3	88	1	8	0	100	88.00
Urban Areas	1	5	86	5	3	100	86.00
Cleared Lands	0	3	7	88	1	99	88.88
Water bodies	0	0	3	0	97	100	97.00
Total	100	99	99	101	101	500	90.88
Producer’s Accuracy (%)	96.00	89.00	87.00	87.00	96.00	91.00	

**Table 4:** Confusion matrix of 2009 land cover classification.

LULCC 2009	Primary	Agricultural	Urban	Cleared	Water	Total	User (%)
Primary Forests	94	4	2	4	0	104	90.38
Agricultural Lands	2	89	5	2	0	98	90.82
Urban Areas	6	8	88	5	0	107	82.24
Cleared Lands	4	1	2	85	0	92	92.40
Water bodies	0	0	1	0	98	99	98.99
Total	106	102	98	96	98	500	90.96
Producer’s Accuracy (%)	97.00	89.00	92.00	95.00	100.00	94.60	

### Land cover change across elevation class

**Fig 3:**
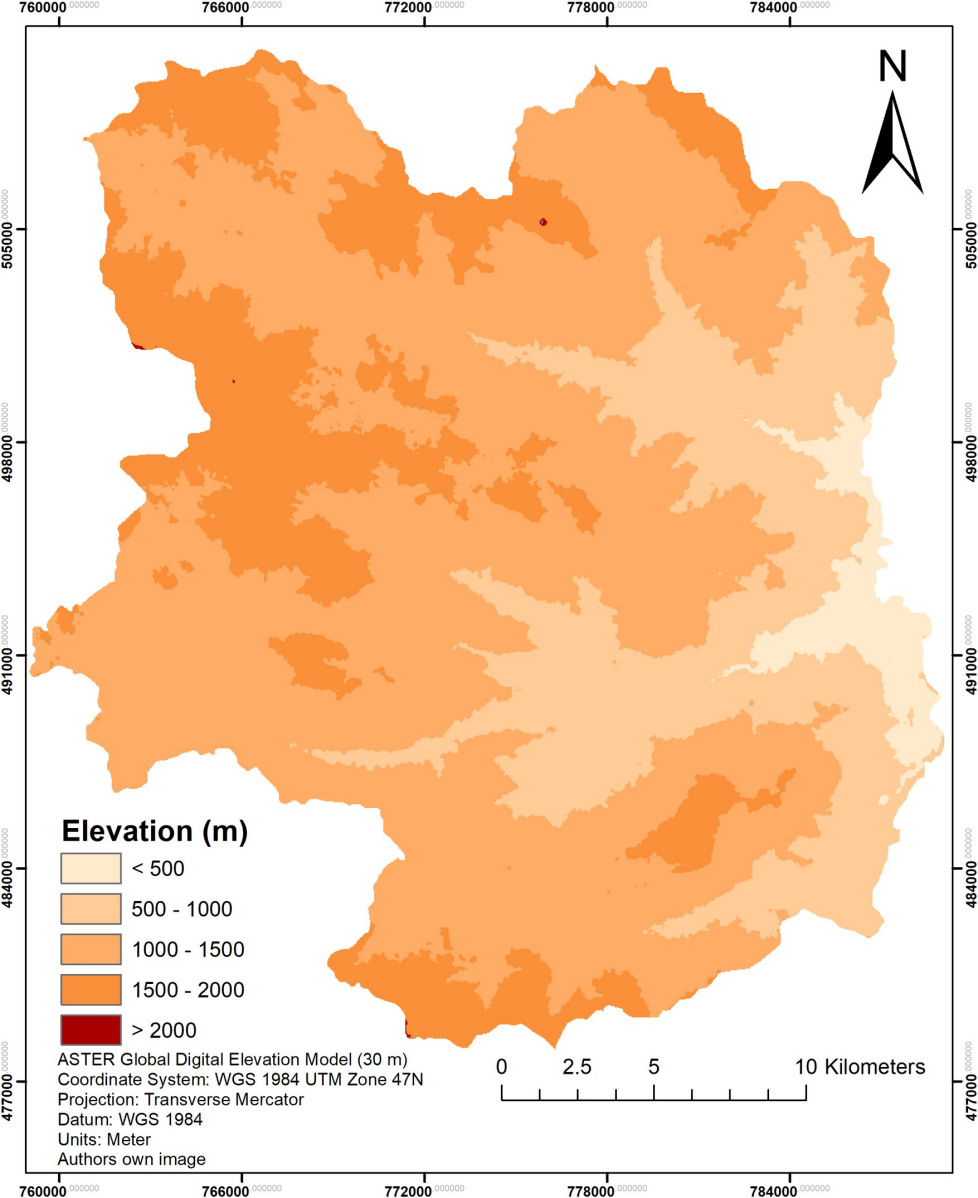
Elevation map of Cameron Highlands. (Data source: ASTER Global Digital Elevation Model courtesy of NASA Earth Data).

**Fig 4:**
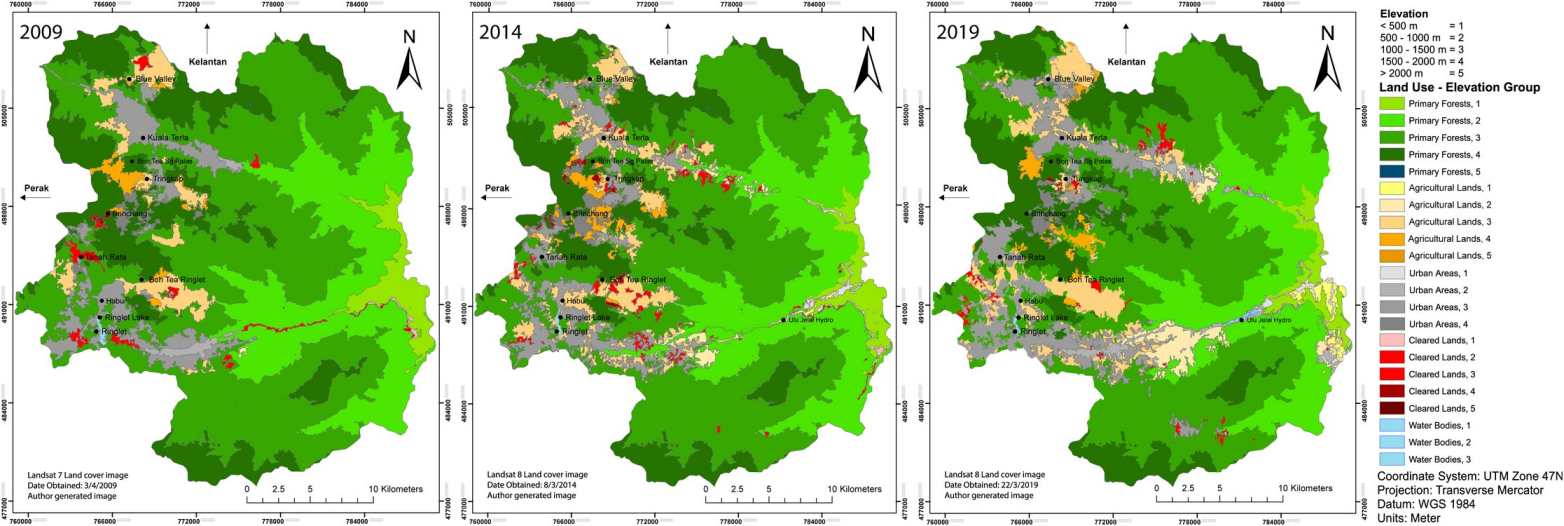
LULCC of Cameron Highlands by elevation group between 2009 and 2019. (Data source: Landsat 7 ETM+ and Landsat-8 OLI image courtesy of the U.S. Geological Survey).

The elevation of Cameron Highlands is categorized by a 500 m interval, shown in Fig 3. In this section, the relationship between LULC classes against elevation gradient is assessed accordingly as seen in Fig 4 and Table 5. Based on the results, it was found that the elevation group of 1000–1500 m experienced the most changes in land cover, where the primary forests declined by 35.98 km^2^ between 2009 and 2019. This reduction in primary forests is followed by an increase of both urban areas and agricultural lands of 3.99 km^2^ and 14.79 km^2^ respectively. As the majority of urban and farm-based plots reside in this elevation group, the changes have been detrimental to the environment in this elevation group. Consequently, land cover classes in the > 1500 m group showed the second largest change, especially in primary forests where urban areas replaced those land covers. During the initial 5 years gap of 2009 to 2014, clearing of primary forests were the main activity alongside agricultural land expansion (farmland and plantations). It was noted that land cover change declined between 2014 and 2019, for all, but urban classes. This is due to the growing urban population and land use where high rise condominiums and buildings, and hilltop homes are being built. As the elevation increases, so does the development of urban living quarters. Interestingly, between 2014 and 2019, agricultural lands experienced a reduction in land cover area. This is due to the conversion of agriculture to urban areas, happening in the 1000–1500 m group. Water bodies such as the Ringlet Lake and Ulu Jelai Hydroelectric plant too, experienced some changes, a decline of 0.22 km^2^ between 2009 and 2019 due to the reduction in size of Ringlet lake, followed by an incline of 0.04 km^2^ for the hydroelectric plant. Based on Fig 3, there are 5 classes of elevation groups, however, the results of land use and land cover classification showed negligible change in the > 2000 m group. Due to the nature of the results, only 2 decimal points are taken for the area. Thus the > 2000 m group has miniscule changes in 3 decimal points; therefore, this group was removed from Table 5.

**Table 5:** LU/LC distribution of area and area change (km^2^) across the elevation (m).

Land Cover	Elevation (m)	2009		2014		2019		2009–2014	2014–2019	2009–2019
		Area	%	Area	%	Area	%	Change	Change	Change
Primary Forests	< 500	21.59	3.73	18.51	3.42	16.93	3.26	-3.08	-1.58	-4.66
	500–1000	132.71	22.95	124.55	22.98	121.59	23.45	-8.16	-2.96	-11.12
	1000–1500	305.23	52.80	286.32	52.86	269.25	51.91	-18.91	-17.07	-35.98
	> 1500	118.62	20.52	112.38	20.74	110.94	21.38	-6.24	-1.44	-7.68
Total		578.15		541.76		518.71		-36.39	-23.05	-59.44
Agricultural Lands	< 500	0.00	0.00	0.49	0.86	7.45	8.80	0.49	6.96	7.45
	500–1000	0.29	0.87	5.46	9.58	23.17	27.39	5.17	17.71	22.88
	1000–1500	25.87	78.40	41.54	72.84	40.66	48.06	15.67	-0.88	14.79
	> 1500	6.84	20.73	9.54	16.72	13.33	15.75	2.70	3.79	6.49
Total		33.00		57.03		84.61		24.03	27.58	51.61
Urban Areas	< 500	0	0	2.51	4.22	2.65	4.33	2.51	0.14	2.65
	500–1000	4.02	8.04	6.45	10.84	4.77	7.81	2.43	-1.68	0.75
	1000–1500	41.78	83.36	43.87	73.73	45.77	74.91	2.09	1.9	3.99
	> 1500	4.30	8.60	6.67	11.21	7.91	12.95	2.37	1.24	3.61
Total		50.10		59.50		61.10		9.40	1.60	11.00
Cleared Lands	< 500	0.67	8.55	0.78	7.05	0.31	7.43	0.11	-0.47	-0.36
	500–1000	1.5	19.12	1.56	14.11	0.19	4.56	0.06	-1.37	-1.31
	1000–1500	5.16	65.82	7.04	63.66	3.29	78.90	1.88	-3.75	-1.87
	> 1500	0.51	6.51	1.68	15.18	0.38	9.11	1.17	-1.30	-0.13
Total		7.84		11.06		4.17		3.22	-6.89	-3.67
Water Bodies	< 500	0.00	0.00	0.00	0.00	0.1	9.09	0.00	0.10	0.10
	500–1000	0.00	0.00	0.00	0.00	0.62	56.36	0.00	0.62	0.62
	1000–1500	0.60	100	0.34	100	0.38	34.55	-0.26	0.04	-0.22
	> 1500	0.00	0.00	0.00	0.00	0.00	0.00	0.00	0.00	0.00
Total		0.60		0.34		1.10		-0.26	0.76	0.50

In Fig 4, the LULCC of Cameron Highlands are grouped by their individual category of elevation–denoted by the values 1–5. This figure enables the reader to have a proper assessment of the land cover changes and expansion between 2009 and 2019. The figure was generated from the results of Table 5. Between 2009 and 2014, the change in urban areas ranged between 2.09 to 2.51 km^2^, while agricultural lands expanded between 0.49 to 15.67 km^2^. Some areas of agricultural lands appeared sporadically as if unplanned, these can be seen in Fig 4, year 2014, occurring in the areas of Habu and Ringlet. These areas seem to be converted from previously urban or primary too, to agriculture. Ground visits to the location further confirms the change as such, particularly in the areas of Brinchang and Tringkap. Notably, most farm areas located along the side of the road network remained unchanged but inland land conversion appeared to be rampant; this further confirms the land use change results to be true to a certain level. The pattern of growth in land use across all classes concentrate their expansion in the fourth elevation group (between 1500 and 2000 m).

### Land cover change across slope class

**Fig 5:**
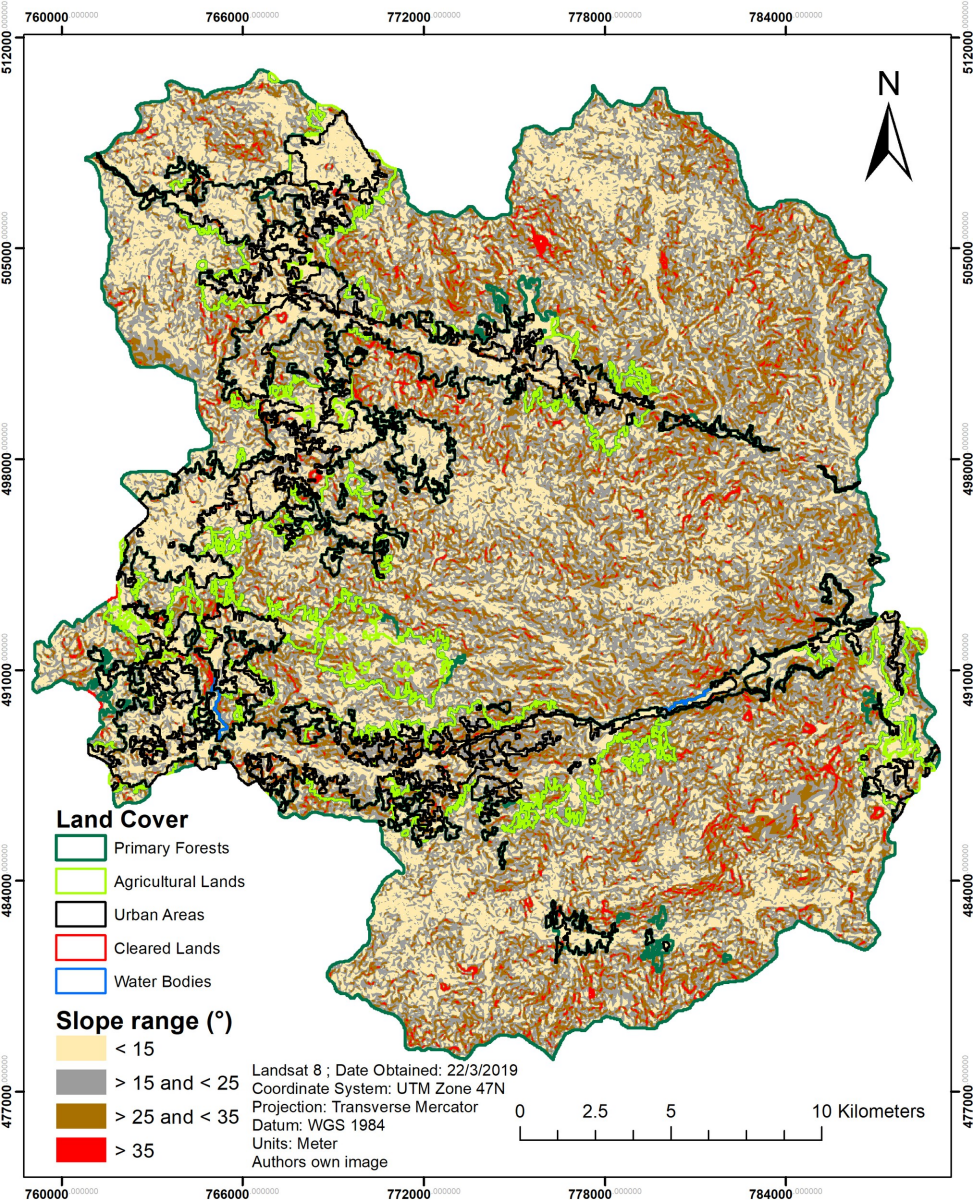
Cameron Highlands slope and land cover image overlay. (Data source: Landsat-8 OLI image courtesy of the U.S. Geological Survey).

According to the Malaysia Ministry of Energy and Nature Resources (MMNER) [49], slope ranges must be controlled for any development activity. Classified details of slope ranges are provided in Table 6. Using these classification systems, all development activities, such as farming, residential construction, and other relevant activities, must follow the guidelines to manage, conserve, and maintain the structures of hill areas. However, this regulation is not applied to the development of most activities in Cameron Highlands. The slope-land use comparison map in Fig 5 provides an indication of the types of land cover that are being built on with regards to the slope inclination.

**Table 6:** Classification of slope ranges [9, 31, 4,9].

Class Names	Characteristic of Slope	Description
Class I	< 15°	Suitable for development, low restriction in geoengineering process, low engineering cost for development and need for research land development.
Class II	> 15° and < 25°	Low restriction in geoengineering process, suitable for development, low engineering cost for development and need for research land development.
Class III	> 25° and < 35°	High restriction in geoengineering process, low in development activities, high cost for engineering development and need for land research development intensively.
Class IV	> 35°	Not suitable for development activities, very high restriction in geoengineering processes, very high cost for engineering development and very intensive need of land research development activities.

In Fig 5, a land cover map of Cameron Highlands in the year 2019 over a slope map in degree format is shown. Areas of steepness above 35° are denoted as areas (in red), wherein any construction or land use is not recommended. Agricultural lands are mostly located in the study area at a slope angle between 15° and 25°. The historical trend between 2009 and 2019 has shown land cover changes to occur primarily between 15° and 25° (Table 7), and second between 25° and 35°; the changes are noted by 25.97 km^2^ and 13.21 km^2^ respectively. However, Fig 5 shows extreme cases in which crops are being cultivated on slopes exceeding 35° in steepness. Our results in 2019 confirm the change in land cover on the upper slopes, where agricultural lands (1.84 km^2^) and urban areas (1.49 km^2^) are the primary land covers in the area. Field visits have confirmed that farmers can be seen cultivating vegetables, typically in “Mossy forest” areas of steep hills at the foots of mountains, while living along the cliff sides.

**Table 7:** LU/LC distribution of area and area change (km^2^) across the slope (°).

Land Cover	Slope (°)	2009		2014		2019		2009–2014	2014–2019	2009–2019
		Area	%	Area	%	Area	%	Change	Change	Change
Primary Forests	<15	190.63	32.97	175.58	32.41	163.71	31.56	-15.05	-11.87	-26.92
	> 15 and < 25	240.73	41.64	226.88	41.88	218.15	42.06	-13.85	-8.73	-22.58
	> 25 and < 35	126.26	21.84	119.56	22.07	117.87	22.72	-6.70	-1.69	-8.39
	> 35	20.53	3.55	19.74	3.64	18.98	3.66	-0.79	-0.76	-1.55
Total		578.15	100.00	541.76	100.00	518.71	100.00	-36.39	-23.05	-59.44
Agricultural Lands	<15	16.37	49.61	24.10	42.26	27.62	32.64	7.73	3.52	11.25
	> 15 and < 25	11.58	35.09	21.86	38.33	37.55	44.38	10.28	15.69	25.97
	> 25 and < 35	4.39	13.30	9.64	16.90	17.6	20.80	5.25	7.96	13.21
	> 35	0.66	2.00	1.43	2.51	1.84	2.17	0.77	0.41	1.18
Total		33	100.00	57.03	100.00	84.61	100.00	24.03	27.58	51.61
Urban Areas	<15	23.01	45.93	30.85	148.32	27.2	44.52	7.84	-3.65	4.19
	> 15 and < 25	19.21	38.34	20.80	34.96	21.16	34.63	1.59	0.36	1.95
	> 25 and < 35	6.88	13.73	6.87	11.55	11.25	18.41	-0.01	4.38	4.37
	> 35	1.00	2.00	0.98	1.65	1.49	2.44	-0.02	0.51	0.49
Total		50.10	100.00	59.50	196.47	61.10	100.00	9.40	1.60	11.00
Cleared Lands	<15	4.79	61.10	4.31	38.97	2.25	53.96	-0.48	-2.06	-2.54
	> 15 and < 25	2.35	29.97	4.37	39.51	1.26	30.22	2.02	-3.11	-1.09
	> 25 and < 35	0.64	8.16	2.13	19.26	0.54	12.95	1.49	-1.59	-0.1
	> 35	0.06	0.77	0.25	2.26	0.12	2.88	0.19	-0.13	0.06
Total		7.84	100.00	11.06	100.00	4.17	100.00	3.22	-6.89	-3.67
Water Bodies	<15	0.33	55.00	0.25	73.53	0.62	56.36	-0.08	0.37	0.29
	> 15 and < 25	0.18	30.00	0.06	17.65	0.36	32.73	-0.12	0.3	0.18
	> 25 and < 35	0.07	11.67	0.02	5.88	0.11	10.00	-0.05	0.09	0.04
	> 35	0.02	3.33	0.01	2.94	0.01	0.91	-0.01	0.00	-0.01
Total		0.60	100.00	0.34	100.00	1.10	100.00	-0.26	0.76	0.5

The results of the land cover to slope classification shown in Table 7 indicate that man-made structures (see agricultural lands, urban areas, and cleared lands) are built between 15 and 35°. Between 2014 and 2019, there was an increase in development at the lower elevations; slope range was not considered here because the majority of the Cameron Highlands, regardless of the slope, has experienced geoengineering processes on its cliff faces. However, a concurrent increase in the lower elevation regions of the study area was recorded.

### Relationship between LST and topography

The mean LST of the Cameron Highlands in the last 10 years increased from 21.1°C to 28.6°C, with an approximate 7.5°C increase across the region (Table 8). As previously mentioned, the LST values are derived from the mean of values from MODIS and Landsat sensors. This was done to minimize the error of deviation and decrease the LST variability difference between the three dates. The primary forest experienced the largest change in LST between 2009 and 2019, where a range of values from -0.5 to 3.5°C are realised. This change is brought about by deforestation and land cover change which occurred significantly in the 500–1000 m group. About 1.7°C increase is observed between 2014 and 2019, while a 3.2°C increase between 2009 and 2019 is observed. The land cover change in this elevation group experienced land conversion from primary forest to agriculture lands and urban areas, predominantly in the areas surrounding the Ulu Jelai Hydroelectric plant, in the East region of Cameron Highlands.

**Table 8:** LST trend per land cover–elevation group.

Land Cover		2009	2014	2019	2009–2014	2014–2019	2009–2019
	Elevation (m)	Temp (°c)	Temp (°c)	Temp (°c)	Change	Change	Change
Primary Forests	< 500	24.1	26.6	27.6	2.5	1.0	3.5
	500–1000	23.4	24.9	26.6	1.5	1.7	3.2
	1000–1500	22.6	24.3	24.8	1.7	0.5	2.2
	1500–2000	22.3	24.0	23.7	1.7	-0.3	1.4
	> 2000	21.3	22.6	22.1	1.3	-0.5	0.8
Agricultural Lands	< 500	-	27.3	26.4	-	-0.9	-
	500–1000	24.5	26.1	26.3	1.6	0.2	1.8
	1000–1500	23.2	25.8	26.2	2.6	0.4	3.0
	1500–2000	22.3	25.5	26.2	3.2	0.7	3.9
	> 2000	21.1	-	25.1	-	-	4.0
Urban Areas	< 500	-	26.6	28.6	-	2.0	-
	500–1000	25.5	26.4	27.9	0.9	1.5	2.4
	1000–1500	24.6	25.8	27.8	1.2	2.0	3.2
	1500–2000	24.2	25.7	27.1	1.5	1.4	2.9
	> 2000	-	-	-	-	-	-
Cleared Lands	< 500	24.9	26.7	27.5	1.8	0.8	2.6
	500–1000	24.4	26.4	27.4	2.8	1.0	3.0
	1000–1500	24.3	26.0	27.2	2.7	1.2	2.9
	1500–2000	23.8	25.3	25.6	2.5	0.3	1.8
	> 2000	-	24.4	-	-	-	-
Water Bodies	< 500	-	-	27.9	-	-	-
	500–1000	-		27.8	-	-	-
	1000–1500	22.3	26.5	27.6	4.2	1.1	5.3
	1500–2000	-	-	-	-	-	-
	> 2000	-	-	-	-	-	-

In the agricultural lands, the 1500–2000 m group elevation is the most affected group by LST change. The increase in LST depends on the type of crop grown. From field visits and verification through Google Earth, the agriculture plots at this elevation group are mainly farm vegetables, flowers, green crops and farms of various vegetative signatures (Fig 4, agricultural lands, elevation group 4). A point to note is the unavailability of LST values in certain elevation groups, this is due to the absence of that land cover class, where a (-) is denoted in Table 5. From the mean temperature difference, it is evident that the mean LULC classes temperature increased over time, thus, indicating that the LST trend in temperature of Cameron Highlands is decreasing.

Aside from assessing LST trends by elevation group, an additional assessment relating to slope inclination was performed. As the Cameron Highlands is an area full of undulating hills and mountains, it is necessary to determine and assess any changes relating to LST and LULC along the slope classes. Using the data from Table 7 and Fig 5, LST values per land cover and slope group are obtained (Table 9). Most of the classes experienced an increase in temperature, while urban and water bodies experienced a decrease in temperature; between 2009 and 2014 for several slope classes.

**Table 9:** LST trend per land cover–slope group.

Land Cover		2009	2014	2019	2009–2014	2014–2019	2009–2019
	Slope (m)	Temp (°c)	Temp (°c)	Temp (°c)	Change	Change	Change
Primary Forests	< 15	22.8	24.4	25.2	1.6	0.8	2.4
	> 15 and < 25	23.0	24.4	25.4	1.4	1.0	2.4
	> 25 and < 35	22.2	24.5	25.4	2.3	0.9	3.2
	> 35^o^	22.5	24.3	25.2	1.8	0.9	2.7
Agricultural Lands	< 15	22.2	23.7	26.6	1.5	2.9	4.4
	> 15 and < 25	22.5	23.5	26.5	1.0	3.0	4.0
	> 25 and < 35	22.7	23.5	25.6	0.8	2.1	2.9
	> 35^o^	23.3	23.5	25.0	0.2	1.5	1.7
Urban Areas	< 15	24.6	25.2	26.8	0.6	1.6	2.2
	> 15 and < 25	24.9	24.8	26.7	-0.1	1.9	1.8
	> 25 and < 35	24.9	24.3	26.5	-0.6	2.2	1.6
	> 35^o^	25.6	24.1	25.7	-1.5	1.6	0.1
Cleared Lands	< 15	24.6	25.4	26.4	0.8	1.0	1.8
	> 15 and < 25	24.3	25.0	26.4	0.7	1.4	2.1
	> 25 and < 35	24.7	25.3	26.2	0.6	0.9	1.5
	> 35^o^	24.8	24.9	26.1	0.1	1.2	1.3
Water Bodies	< 15	21.1	23.3	25.6	2.2	2.3	4.5
	> 15 and < 25	21.6	23.8	25.5	2.2	1.7	3.9
	> 25 and < 35	23.5	23.7	24.7	0.2	1.0	1.2
	> 35^o^	25.4	23.2	24.4	-2.2	1.2	-1.0

### Significance of LULCC and topography

To assess the significance of LULCC from Fig 4 and Tables 5 and 7, the ANOVA test showed that the changes were significant for primary and agricultural lands, and urban areas, although the changes according to the forest elevation and slope classes illustrated otherwise (Table 10).

**Table 10:** Non-parametric ANOVA test of significance on land cover changes comparisons.

Land Cover vs Elevation		Land Cover VS Slope	
Land Cover classes	P-values	Land cover classes	P-values
Primary Forest	0.0002	Primary Forest	<0.0001
Agricultural Lands	0.0023	Agricultural Lands	<0.0001
Cleared Lands	0.9869	Cleared Lands	0.9789
Urban Areas	0.0074	Urban Areas	<0.0001
Water Bodies	0.9770	Water Bodies	0.9566
Elevation	P-values	Slope	P-values
< 500	1.0000	< 15	1.0000
500–1000	0.9334	> 15 and < 25	1.0000
1000–1500	0.7372	> 25 and < 35	1.0000
> 1500	0.8003	> 35	0.9990

### Significance of LST and topography

In the significance test of LST from Tables 8 and 9, the ANOVA test showed that the changes were significant for water bodies only when land cover against elevation was analysed (Table 11). Elevation groups 1 and 2 too were significant. However, land cover against slope classes achieved significance across primary forests, agricultural lands, and cleared lands. The LST change in the three slope classes, excluding > 35°, were significant too.

**Table 11:** Non-parametric ANOVA test of LST significance on LULC–topography.

Land Cover vs Elevation		Land Cover VS Slope	
Land Cover classes	P-values	Land cover classes	P-values
Primary Forest	0.5416	Primary Forest	0.0100
Agricultural Lands	0.0604	Agricultural Lands	0.0044
Cleared Lands	0.4768	Cleared Lands	0.0077
Urban Areas	0.0779	Urban Areas	0.0123
Water Bodies	0.0100	Water Bodies	0.0199
Elevation	P-values	Slope	P-values
< 500	0.0012	< 15	0.0047
500–1000	0.0064	> 15 and < 25	0.0049
1000–1500	0.0349	> 25 and < 35	0.0032
1500–2000	0.5538	> 35	0.0245
> 2000	0.7572		

## Discussions

### Driving forces of land cover change and effects on land use

In the last ten years, an extensive body of literature and theories has emerged, wherein land use and land cover change are directly related to forest transition. Drivers of deforestation are identified based on permanent changes of forest cover to other land uses. The land use changes described in this study indicate the growth of agriculture and urban expansion through the clearing of previously used land and a new land—deforestation and forest conversion [50, 51]. This research revealed significant changes in both spatial composition and configuration due to urban development as a result of the land use structure in the study area. In Cameron Highlands, this study found that the main contributors to land use change are driven by a rise in agricultural plantations and the agricultural activity of vegetable farming. Subsequent drivers of deforestation include urbanization, for which tourism and living spaces are in demand. This has led to further deforestation and forest conversion to meet this national demand. In hindsight, this change is also seen in other parts of the world where urban growth far exceeds the supply of food sources [18, 23]. Hence, land use change in Cameron Highlands shifted from mostly primary forest area in 2009 to mostly urban areas in 2014 and, subsequently, agricultural lands in 2019. Similarly, Gasim et al. [13] and Razali et al. [29] reported that human activities and increased demand for both forest products and arable land for agriculture are the primary drivers of deforestation in the study area. Despite the decrease in primary forested areas, we can also observe some new growth in tea plantations and vegetable crops.

The main driving factor is undoubtedly deforestation, but there are also various proximate drivers. It has been observed that the intensification of agricultural expansion, settlement expansion, human activities, forest product harvesting, and mining are key contributors to land use in Cameron Highlands (Fig 4). There are also the factors of government policies on the expansion of agriculture to eliminate poverty in the region; human population growth; and advancements in plantation technologies, arable soil, and climate suitability, which are the various underlying drivers of land use changes [6, 29, 35].

Population loss also plays a role in a regional growth structure by means of economic as well as land use management. You et al. [52] expands on his research in Northeast China during their economic boom era of 1992 to 2018. It was found that the reduction in population in the region led to a decline in both social welfare and a reduction of socioeconomic development. This had shifted the transitional land use from farming to a more industrialised sector in hope to bring the migrants back. With regards to the farming systems and technological advances, we are able to see a decline in local communities in Cameron Highlands that have been solely concentrating on farm development [6]. The shift is being made to a transformation of economic rise in the industrial plantation and tourism sector [6, 31]. However, such change results in the increase of foreign investors buying up local owned land, which inadvertently pumps our homegrown income out of the country [31]. Many aspects based on the local population’s perceptions of land use, their socio-economic conditions, farming systems and technologies, local environmental policies, and the role of politics remain as challenges for land planners, as these aspects have influenced the changes that occur on the forest cover [53].

### Land use/ land cover changes relationship with topography

By taking the topography–LULC relationship into consideration, our study observed that the LULC along the upper angles exceeds 35°. Additionally, LULCC was found to be highly dependent on the slope, elevations, the geological structures of terrains, and environmental–ecological circumstances. Because agricultural activities prevail in predominantly flat areas, this result was expected due to the ease of agricultural practices. It is clear that flat areas are more conducive to agricultural practices such as irrigation and ploughing, allow a higher intake of soil nutrients due to low surface runoff, and have close proximity to human settlements in which grazing occurs [18, 54]. In the higher elevation region of the Cameron Highlands (1500–2000 m) in 2019, we found that the area of urban areas was greater than the area of primary forests. This decline in primary forest is also attributable to the rise in farmland, which caused instability in the soil in the surrounding area. Despite the high risks of soil erosion, further expansion occurs annually, to the detriment of farmers; annual soil deposition is found to occur at a rate of 0.5–1.0% of the total soil area (Huon et al., 2017). According to Huon et al. [55], in Laos, this rate of soil deposition is calculated based on an area that has high rainfall and is on a steeply sloping plane. The study area in Laos is highly similar to that of our study area of the Cameron Highlands. In the Lancang–Mekong river delta, Chuenchum et al. [56] found that the rate of deposition varies at different elevations and distances of the affected area from the closest water table and/or river. The erosion rate was found to be between 700 and 10,000 t/km^2^/y in the Mekong river delta. However, in our study, the rate is approximately 90 to 200 t/km^2^/y, depending on the area [35]. Sungai Bertam is not a fast-moving river, and its rate of flow is controlled continuously by the Ulu Jelai hydroelectric plant that flows into Ringlet Lake. Thus, the amount of annual rainfall, in addition to the ongoing engineering projects in the river bed at Ringlet, constantly causes the rate of soil deposition to flux, particularly during the monsoon season [35].

Generally, farmland is most suitable for production between slopes of 15 and 35°. However, the results in Table 7 show a continuous rise in farmed land above 35°. This is a cause for concern because steeper slopes not only have higher development costs due to their inaccessibility and fragile soil structure but also lower soil fertility due to faster surface runoff, poor water holding capacity, and weaker root hold strength for crops. Although areas for farming and agricultural activities are not scarce, the uncontrolled growth of crops appears to violate the regulations because these crops are planted on cliffs, regardless of the slope degree and elevation. This has significant implications for the cliff face, increasing the risk of potential ground imperfections and the formation of loose soil, thus leading to landslides [25].

Additionally, this practice leads to unforeseen effects caused by the uprooting of previously grown trees with deep roots, which are replanted with soft root crops [57]. Unlawful farming operations are to blame for this phenomenon due to the placement of farmlands on the upper classes (III and IV) of hillslopes. Agriculture development on these hillslopes directly affects the water quality of streams, through which soil degradation is the direct cause of improper farming guidelines [58]. Several studies by Hamzah et al. [31], Birhanu et al. [59], and Degife et al. [60] reported that flat land is more favorable than steep slopes due to a lower water table, better water holding capacity, and maximum contact time of the seepage of water into the soil. As the majority of flat lands and low degree cliff faces are used, local people will choose to deplete the primary forests at higher elevations and on steeper slopes. Further development will be pushed into the lower regions of the Cameron Highlands, primarily in the hill dipterocarp region (500–1000 m) and areas in the upper dipterocarp region (1000–1500 m); thus, further increasing the rate of deforestation and leading to a heightened change in climate processes.

Generally, primary forests decrease with an increase in agricultural land, despite the elevation gradient and high angular slope degree, which is partly caused by the conversion from forest to farmland or urban areas to farmland. The Cameron Highlands has experienced numerous landslides and cliff failures in recent decades, primarily due to poor geotechnical practices [61]. However, these occurrences were caused by the uprooting of trees from these steep slopes, which is one of the detrimental effects of cliff face deforestation [15]. This, consequently, leads to an increase in surface water runoff and exacerbates the effect of soil erosion due to the absence of vegetation cover [16]. Because of the increased changes due to anthropogenic factors and their resulting impacts, recording and addressing these issues is of extreme importance.

### Topographical relationship to LST and LULCC

Most of the data obtained for the calculation of LST are within the first quarter of the year, when the monsoon season is faltering or has passed. It can be noted that, during this time, the monsoon season affected the data because the land is usually covered with damp vegetation, which results in a cooling effect. In contrast, at the end of the monsoon season towards summer, the vegetation cover was minimal; hence, the recorded LST for most years was high. For every 100 m rise in elevation, the surrounding temperature decreases by 0.65°C, depending on the location, season, water vapor content, time of day, and various other factors [50]. Using this classification of elevation against recorded LST, we were able to determine the extent to which the constant rate of 0.65°C per 100 m increased during the period of 2009 to 2019.

Both elevation groups one and two experienced the most change in LST in the span of 10 years. It was observed in primary forests and cleared lands by a change of 3.2–3.5°C and 1.8–3.0°C, respectively. When the tropical rainforests were deforested, they would have released carbon to the atmosphere which affects the surrounding area. This area is found to be in the Eastern part of the Cameron highlands region where both agriculture and urban classes have taken over. It can be noted that a road was built here between 2009 and 2014, linking the Eastern and Western boundaries together. Subsequently, urban development increased tremendously in the later 2014–2019 years. Although there are no urban housing areas evident, as validated by Google Earth and Maps, the area in the lower elevation region seems to be developed for farm workers. While it is difficult to ascertain whether the area is solely urban or urban mixed–agriculture, we are able to confirm that land use change has severely increased the overall LST.

In the third elevation group (1000–1500 m) lies the upper dipterocarp region; Liang et al. [62] and Sinha et al. [63] explained that an increase in the sensitivity of forests to changes in climate conditions begins here, where the sensitivity of the forest flora increases as the elevation increases. The species biodiversity richness is greater here, and flora is able to thrive better than in other forested regions because the climatic conditions and the atmospheric pressure are optimal. However, from our results, we have found that LULCC in this elevation group is exceptionally higher than other elevation groups. Because of the growing population, the demand for land for urbanization in the Cameron Highlands will continue in the already populous third elevation group. Furthermore, because available land is becoming scarce, future urbanization can be expected to occur in the lower forest region (first and second elevation groups). Moreover, the change in LST across all land covers is significantly higher than the other elevations (Table 8). It is interesting to note that water bodies had an increase in temperature by 4.2°C, 1.1°C, and 5.3°C in 2009–2014, 2014–2019, and 2009–2019. One of the water bodies’ is named as Ringlet Lake and has experienced land cover changes between 2014 and 2019, where some areas were filled and converted to aquaculture farms. This change in land use suggests that the increase in temperature is justified where the surface reflectance of water has now increased while the rate of heat deposition has decreased. As the aquaculture farms have aluminium roofing, the heat absorption in this area increases as well.

The fourth elevation group (1500–2000 m) lies the montane-forest oak region. It is regarded as the region of utmost importance in the entire Cameron Highlands area, due to the majority of tea plantations and farm crops grown there. Hence, results for this would indicate the suitability of crop growth for the future. From our results, the average change in temperature was noticeably higher in 2009–2014, than 2014–2019 for all classes. This result would indicate that the intensive LULCC happening between 2009 and 2014 affected the LST severely; a noticeable change between 1.5–3.2°C. The results obtained indicated a similarity to those of the study of Phan et al. [64], which was conducted in a mountainous region of Vietnam when both MODIS and Landsat datasets were averaged as well. Rising temperatures not only cause hardships for agriculture but also negatively impact the tourism sector [64–66].

### Relationship between LST and LULCC

Changes in land use had affected the land surface temperature (LST) of the Cameron Highlands, which is also a driver of forest transition. The relationship between LULCC and LST is complex and multidirectional as land use change has been demonstrated to influence climate at local, regional, and global scales. Among anthropogenic land use types, the urban environment and urbanization are arguably the strongest drivers of localized climate change and variability [67]. In Cameron Highlands, preliminary research showed a rise in temperature by roughly 2–3.5°C in ten years [35]. This increase was introduced by the change in land cover and extreme land use conversion of forest to urban and from urban to agriculture. The changes occurring in major towns such as Ringlet, Brinchang, and Kuala Terla are readily observable [35]. Moreover, the increase in urban areas is a direct indication of an increase in population, which affects the urban heat index (UHI). Furthermore, the conversion of the vegetative regions to impervious surfaces has been shown to increase extremes in temperature and result in the creation of urban heat islands (UHI). Urban development and construction activities were demonstrated by Madanian et al. [68] to result in alterations in climatic parameters, such as land temperature. The loss of tropical forest and its replacement with intensive monoculture has also been demonstrated to result in localized and regional alterations to the climate [69]. Parks in urban areas mitigate the excess heat radiated off buildings by providing a cooling effect from the land cover types of water and vegetation. However, not much is heard about the effects of buildings on those parks and how the excess heat would affect the cold island effect (CIE). As mentioned before, UHI is contributed by urban development, hence, Han et al. [70] studied the impacts of buildings and their UHI against cool areas such as urban parks. This study was done in Beijing, China in multiple central locations where urban parks have been situated to mitigate the UHI effects from the close proximity of buildings. The results have shown the parks CIE to be stronger in summer than in winter. Moreover, high rise buildings would provide shade onto the park areas, which in hand cools the area by a nominal amount. However, as high-rise buildings occupy a larger surface area, it too is able to radiate more heat from the sun. Han suggests that the planning of an urban city should take into account the effects of medium and high-rise buildings onto parks. In Dalian city, China, Yang J et al. [67] had organized and planned local climate zones (LCZ) for the mitigation of urban heat island effect. In such a densely populated city, human settlements are bound to generate excess heat, further contributing to the increment of the overall LST. Through a series of models, an appropriate LCZ layout model was found achieving 11.654°C, as the lowest UHI intensity value. This layout model is recommended for similar densely populated urban areas [26]. Similarly, Yang J et al. [71] had studied the relationship between LST and LCZ in Shenyang city, China using these parametric models too. Notably, this LCZ model would be beneficial to the urban development of Cameron Highlands and to understand the severity of UHI emitting from these areas.

Studies in Cameron Highlands over the past years have shown an inclination in LST due to the direct implications of forest clearing—even more so with the extensive growth of urban housing areas [29]. In forest transitions, it has a very sensitive relationship with the changing climate. Modelling studies focusing on the Amazon basin established that a loss of biodiversity and increase in the savanna would result in ecological “tipping points”—namely, an increase in temperature of 4°C or deforestation exceeding 40% of the forest area. This would cause a ‘runaway’ effect in which large-scale transitions of mostly the southern and eastern Amazon could take place [69]. As of 2016, an estimated 1°C temperature increase has been observed in the Amazon region [69]. Changes in climate due to land use behaviours and greenhouse gas emissions could also potentially influence the hydrological regimes of geomorphological features such as rivers catchments and basins, as well as affecting the run-off process [68, 69]. Patterns of vegetation types in relation to LST is studied in Coastal Dalian using the MODIS12Q2 product, where each vegetation phenology identified by NDVI was found having a strong relationship against LST [72]. Results have shown the LST in these coastal areas affecting the vegetation cycles, primarily to the contribution of UHI from nearby urban areas. The results of a study conducted in Isfahan Province, Iran utilized a Landsat-based analysis to show a strong negative correlation between LST and the Normalized Difference Vegetation Index (NDVI) during hot months. Conversely, the study showed the opposite during the cold season. This phenomenon was also demonstrated by assessing changes in vegetation cover, moisture properties, and surface temperature in a brown coal dump from 1984 to 2009 using a Landsat-based analysis [73]. By comparing Fig 4 to Table 8 and Fig 5 to Table 9, the changes in land use difference over the years were also shown to severely impact the average recorded LST. As this is the first study to associate LST with forest type cover class in Malaysia, we hope that this work can serve as a starting point for future studies. The benefit of assessing LST is that LST can serve as a variable to assess forest health; moreover, the LST provides valuable information to understand better what occurs at each elevation and what the LST threshold is, to identify the ecology linked to that LST.

### Sustainable Development Goals (SDG) for Cameron Highlands

The main issues that have affected the Cameron Highlands are the causes and effects of its land-use. The Cameron Highlands is unique because it is a highland forest whose year-long temperature and cool climate provide the perfect grounds for highland vegetable crops and tourism. The expansion into this sensitive highland region is driven by these factors, rather than timber logging. These issues include the transition from small-scale shareholder agriculture to larger scale deforestation through logging. Ideally, the development of the policies in Cameron Highlands would be aimed at mitigating carbon emissions from the deforestation of primary forests, which are great carbon storages. The national sustainable development goals (SDG) outline included estimates of the activity data of land use via satellite land monitoring systems through land cover assessments, increased enforcement funding, and educating the locals about proper farming techniques to ensure sustainable farming practices are undertaken [74–76]. The situation in Cameron Highlands differs from that in other hotspots, such as Borneo, as there are no large-scale industrial plantations or large agriculture plots in Cameron Highlands but instead small-sized areas of agriculture run by many small shareholders. Based on a visual interpretation from visits to the study area, the area’s land usage is improper, as regulations are improperly enforced. Some larger agriculture plots, such as the Bharat tea plantation and Boh tea plantation, may benefit from the SDG’s by furthering the growth of their tea sustainably through planting only on allocated land without pushing into other land-use areas and also following the guidelines for planting on the recommended slope degree class [68].

There are several ways that the government can address the existing drivers of deforestation. First, both economic and employment opportunities for the local and indigenous communities should be expanded. Secondly, there are two groups of drivers for deforestation, the first being the large shareholders and the second being the small communities farming for their own needs [77]. For the first group, the government could strategize and create policies to develop a transparent and effective enforcement mechanism [78]. It is recommended that the authorities in Cameron Highlands establish an incentive mechanism for the second group by rewarding them for their efforts in conserving the forest by means of compensation [78, 79]. However, this can only work in the short-term for such individuals, as they could come to rely on handouts and, in the worst case, this factor could create more unemployment [80].

A baseline for measurements would need to be established, which would either follow the national forest reference value or the emission level. This system would periodically monitor and measure carbon emissions and storage values and provide the information required for periodical reporting to the UN and other relevant national committees. The policies governed by these countries should encourage mitigation actions in the forest sector by reducing emissions from deforestation and forest degradation, engaging in conservation and the enhancement of forest carbon stocks, and conducting sustainable management of forests [74, 81]. The state government of Pahang has announced efforts for the protection and preservation of the Cameron Highlands, similar to the recommendations stated above, including the regrowth of trees and allocating funds to increase armed security forces to curb illegal land encroachment [82, 83]. An additional suggestion to consider is the use of identification and mapping to construct a simple cause-effect relationship chart between the different land uses and their associated socio-economic activities. Through this program, we would be able to specifically establish the pros and cons of an activity and identify the relationship one activity would have with another [84]. This recommendation would be crucial for identifying the causes and effects that would then determine the course of action in Cameron Highlands.

### Limitations of the study

In our study, we first encountered several issues regarding the derivation of the LST from both Landsat 7 and 8 datasets, primarily due to the errors mentioned previously. To counter this issue, we had combined both MODIS and Landsat LST datasets together to form an average. This resulted in the temperature data analysis conducted in this study to be indeed helpful. In our preliminary study by How Jin Aik et al. [35], to counter the uncertainty of results, the two satellite sensors were compared with air temperature obtained from the local meteorological weather station in Tanah Rata, Cameron Highlands. By taking into account the underestimation of air temperature data, we conducted an accuracy assessment based on the projected LST values. The RMSE and BIAS values were obtained through the comparative LST assessment, which was backed up by previous studies of similar land cover and temperate climates, to confirm that the RMSE accuracy is within acceptable range. We had chosen the Landsat sensor due to its long-term date coverage which our study falls within, and compared against MODIS, and air temperature values. Perhaps, the need of another LST derived dataset would result in a better comparison of interannual temperature variability.

In Malaysia, an average of 80% cloud cover is experienced daily [85, 86]. Moreover, the study area sits on the Titiwangsa Range, which separates the north-eastern and south-western part of Malaysia. As the range has an elevation of 1800 m and above, it acts as a natural barrier to protect the southern parts of Malaysia from the harsh monsoon season that batters the north. The date of our study was in late March to early April, this is the period when the northern monsoon had just passed. Even though the temporal resolution of MODIS is 2 days, rains are constant, even at the end of the monsoon season, hence, we would not be able obtain clear cloud coverage images; having a 16 vs 2-day temporal resolution does not help much. This is where radar based LST methods would work. For future studies, we recommend examining the slope effect that determines the lapse rate at each slope range. This is due to the different forest types having different tree canopy heights and surface canopy areas; hence, there would be a difference in the direct radiation received. Moreover, future improvements, such as applying several other land surface temperature detection methods, should be used—for example, LiDAR through UAVs, and AVHRR radar derived LST—to create a more accurate database over the period of study.

## Conclusions

The forests in Cameron Highlands have witnessed an unprecedented rate of deforestation since the 1960s, which accelerated as industrialization and the need for urban growth expanded in priority over conservation. We found the main conclusions as follows: (1) Significant LULC changes were observed in the study area through an analysis of land use vs topography. In a span of 10 years, the agricultural lands and urban area classes increased substantially by 51.61 km^2^ and 11.00 km^2^ respectively. In the primary forest class, the elevation most affected by this change was the 1000–1500 m group. Between 2009 and 2019, it experienced a reduction of 35.98 km^2^ or 5.14% of the total land area. (2) Landscape of land cover along slopes shown agricultural lands pushing into slopes beyond 25°. Moreover, between 2014 and 2019, a recorded land cover change of 1.84 km^2^ and 1.49 km^2^ for agricultural lands and urban areas respectively was found exceeding 35°. (3) In the urban land areas of Cameron Highlands, predominantly in the third and fourth elevation groups, a rising trend of land surface temperature was observed. Within the span of the ten years, temperatures rose by an average of 3°C and an overall 7.5°C is recorded. (4) We have identified the drivers of deforestation in the study area. It is mostly related to the rise in urbanization as a result of population growth and agriculture growth. Therefore, it is essential to have a broader understanding of historical trends and identify the past drivers of deforestation that lead to LULC changes. In this study, we hope that the research methods designed are able to be evaluated to a level where both ecological and social systems can support the green development of the region and approach the target of achieving a low carbon credit value.
